# Interprofessional teaching and learning in the health occupations – A conference developed by and for young scientists in the field of education

**DOI:** 10.3205/zma001149

**Published:** 2018-02-15

**Authors:** Heike Wild, Ina Thierfelder

**Affiliations:** 1 Charité - Universitätsmedizin Berlin, corporate member of Freie Universität Berlin, Humboldt-Universität zu Berlin, and Berlin, Institute of Health Institut für Gesundheit und Pflegewissenschaft, Berlin, Germany

## Introduction

Issues of interprofessional teaching and learning in the education of practitioners in the health occupations are being increasingly addressed from a theoretical as well as an empirical perspective in Germany. To date, the scientific community involved in research on inter-professional teaching and learning in the field of health occupations is still relatively small and poorly connected. This is particularly the case for young scientists who work on relevant topics as part of their bachelor’s, master’s or doctoral theses. Against this background, networking beyond the scope of single disciplines or institutions gains in importance, particularly as a means of building mutual support platforms. This idea motivated us to create a conference that aims to bring together young scientists in the field of interprofessional teaching and learning in Germany for the first time. Scientists working on their habilitation, doctoral, or master’s theses were given the opportunity to engage in a scientific discussion of the challenges posed by interprofessional teaching and learning in the health occupations. Moreover, they were able to present and receive feedback on their research projects in workshops with both peers and expert participants. Apart from providing young scientists with individual support, the conference aimed to identify common interests beyond the scope of single institutions or individuals and to stimulate the establishment of interest groups, in order to engender a scientific national discourse on interprofessional teaching and learning in the field of health occupations. A grant application for financial support for the conference was submitted to the Federal Ministry for Education and Research (Guideline for the support of young scientists in the field of educational research through events). A positive decision on this application made the conference possible and enabled 40 young scientists from all over Germany to meet on the 16^th^ and 17^th^ of November 2017 on the island of Schwanenwerder in Berlin. 

## Program

The main part of this conference organized by the Institute of Health and Nursing Science of the Charité - Universitätsmedizin Berlin consisted of three research workshops. Each workshop included four working sessions and provided a platform for intensive discussions moderated by Prof. Ursula Walkenhorst (University of Osnabrück), Prof. Marion Huber (University of Applied Sciences Zürich) und Dr. Cornelia Mahler (Ruprecht-Karls-University of Heidelberg). Twelve young scientists presented their research projects during the workshops. The presented projects had been previously selected based on specific criteria following a call for abstracts. Depending on the working stage of each project and on participants’ individual counselling needs, methodological issues, the specific topics being researched, as well as issues of transfer from theory into practice were discussed. The selected projects covered a broad spectrum of topics, such as the requirements for educators involved in interprofessional teaching; the development, testing and efficacy of (online) interprofessional teaching and learning formats for basic and continuing education; peer-assisted learning as an approach to interprofessional education; stereotypes endorsed by educators and trainees in the health occupations; students’ attitudes to interprofessional cooperation; and the analysis of setting specific interprofessional communication processes. Various qualitative and quantitative study designs, as well as psychological, pedagogical, sociological or linguistic theories were used to address the above mentioned topics. 

Apart from critically reflecting upon the presented research projects, the conference also aimed to identify networking potential. As a result, a basis for individual as well as institutional cooperations could be established. Moreover, the need for a stronger link between educational theory and practice was discussed.

Two keynote addresses provided the frame for the three research workshops. Prof. Ursula Kessels (Free University Berlin) gave a presentation on heterogeneity as a challenge in interprofessional teaching and learning contexts from the perspectives of social, work and organizational psychology and thus emphasized the need for multidisciplinary research on interprofessional practice. In his presentation, Prof. Martin Fischer (Ludwig-Maximilians-University of Munich) first described the concept of interprofessional cooperation from a philosophical viewpoint, and then gave an overview of the current state of basic and continuing interprofessional education in the health professions.

## Conclusions and Outlook

Overall, this type of conference received very positive feedback. The small number of participants as well as the constructive and supportive atmosphere during the research workshops were very much appreciated. The opportunity for intensive discussions at varying stages of the research process was deemed very valuable and necessary, as science can only move forward through dialogue. Participants particularly highlighted the fact that the conference was the result of a peer initiative. Organizing the conference on a regular basis and its affiliation to the Inter-Professional Committee of the German Society for Medical Education (GMA) were recommended. Future steps include exploring options to provide similar opportunities in the long term, not only to young scientists in the field of interprofessional education, but also to those involved in providing basic and continuing education in the field of health occupations. The results of the conference will be published in a conference proceedings volume (estimated publication date - February 2018).

## Competing interests

The authors declare that they have no competing interests. 

